# SBP Is Superior to MAP to Reflect Tissue Perfusion and Hemodynamic Abnormality Perioperatively

**DOI:** 10.3389/fphys.2021.705558

**Published:** 2021-09-14

**Authors:** Jie Sun, Jing Yuan, Bin Li

**Affiliations:** Department of Anesthesiology, Zhongda Hospital, Southeast University, Nanjing, China

**Keywords:** SBP, MAP—mean arterial pressure, intraoperative hypotension, organ damage, patient outcome

## Abstract

Many articles have reported that intraoperative low mean artery pressure (MAP) or low systolic blood pressure (SBP) impacts on organs’ function and patients’ outcomes perioperatively. On the contrary, what type of blood pressure should be obtained still needs to be clarified. In our paper, we compared the influencing factors of MAP and SBP, and mathematical formula, arterial pulse contour calculation, and cardiovascular physiological knowledge were adopted to discuss how blood pressure can effectively reflect tissue perfusion and hemodynamic abnormality perioperatively. We concluded that MAP can reflect cardiac output change sensitively and SBP can reflect stroke volume change sensitively. Moreover, SBP can reflect the early hemodynamic changes, organs’ perfusion, and heart systolic function. Compared with MAP, perioperative monitoring of SBP and timely detection and treatment of abnormal SBP are very important for the early detection of hemodynamic abnormalities.

Research on the effects of intraoperative hypotension on organ injury and patients’ outcomes began in the 1950s. Fred Wasserman et al. (1955). reported 25 cases of postoperative myocardial infarction and discovered that intraoperative blood pressure decreased more than 40/20 mmHg was one of the risk factors. Monk et al. (2005) proposed that intraoperative systolic blood pressure (SBP) lower than 80 mmHg was an independent risk factor of mortality within 1 year after non-cardiac surgery. Goldman and Caldera (1979) found that decrease of SBP (more than 33% from baseline for more than 10 min) was associated with increased perioperative complications in hypertensive patients. In another large-scale retrospective study of more than 5, 127 patients, Sun et al. (2015) discovered that acute kidney injury could be caused by intraoperative mean artery pressure (MAP) by less than 60 mmHg for 20 min or less than 50 mmHg for 10 min. Most studies suggested that low MAP or SBP impacted on organ function and patient prognosis. However, there is no consensus on the extent of low MAP or low SBP that will cause organ hypo-perfusion or poor patient outcome. The reasons may be that blood pressure is an indirect reflection of blood flow and hypo-perfusion is the cause of organ damage, rather than hypotension. What pressure is more closely related to organ blood flow and tissue perfusion? Is it MAP or SBP? We will discuss this in the following sections of this paper.

## The Relationship Between Map and Cardiac Output (CO)

Since CO is a real flow parameter and flow rate determines tissue perfusion, we have to clarify the relationship between blood pressure and CO. Meng et al. (2018) figured out in their review article that blood pressure did not represent CO and blood flow. CO may be significantly different in the presence of the same MAP. As a result, the pressure threshold that influences CO and organ perfusion may differ between individuals. The reasons are explained as follows. Systolic and diastolic blood pressure can be measured by auscultation method or oscillation method. The MAP can be estimated with the empirical equation (Hertzig et al., 2011):


(1a) MAP=DBP+1/3×(SBP-DBP)

However, for direct intra-artery blood pressure measurement, MAP is calculated with the area under the curve (AUC) in the invasive pressure contour with the equation: MAP = AUC/cardiac cycle. Mathematically, blood flow is described by Darcy’s law (which can be viewed as the fluid equivalent of Ohm’s law) and approximately by Hagen-Poiseuille equation. According to the following relationship, the mean arterial pressure (MAP) is determined by CO, systemic vascular resistance (SVR), and central venous pressure (CVP) based on the relationship among flow, pressure, and resistance: MAP-CVP = CO•SVR. CVP is usually close to 0 mmHg, so this relationship is often simplified to the below equation (Mayet and Hughes, 2003):


(1b) MAP=CO•SVR⁢or⁢MAP=SV•HR•SVR.

If SVR is stable, it is obvious that CO is proportional to MAP. Consequently, MAP can be used to evaluate CO and tissue perfusion. That is why some studies revealed that an MAP of about 60 mmHg was the critical low limit of organ perfusion. When MAP is more than 60 mmHg, increasing the level of MAP can lead to better organ perfusion particularly (Hollenberg et al., 2004). Similar studies had also proven that the recovery of cerebral blood flow, especially microcirculation blood flow, is closely related to MAP (Ristagno et al., 2008). Although MAP and CO are closely related with each other, MAP is not equal to CO. Only when the value of SVR remains unchanged is MAP is proportional to CO (SV•HR).

## The Relationship Between SBP and CO

**FIGURE 1:**
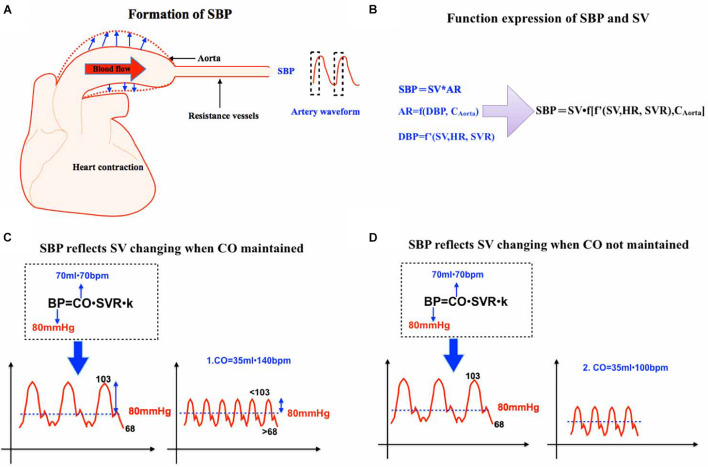
**(A)** This is a schematic diagram of SBP formation. SBP begins with heart contraction and is proportional to SV and aortic resistance. **(B)** Function expression of SBP and SV. **(C)** SBP reflects SV changing when CO is maintained. SV 70 ml, HR 70 bpm, and MAP 80 mmHg is initially assumed. When SV decreases to 35 ml and HR to 140 bpm. If SVR is unchanged, MAP will remain unchanged but SBP will decrease because MAP gets closer to SBP with increased HR. **(D)** SBP reflects SV changing when CO is not maintained. SV 70 ml, HR 70 bpm, and MAP 80 mmHg is initially assumed. When SV decreases to 35 ml and HR only to 100 bpm, both MAP and SBP will significantly decrease. SBP, systolic blood pressure. SV, stoke volume. CO, cardiac output. SVR, systemic vascular resistance. HR, heart rate.

According to Equation 1b, CO is closely related to MAP and, based on Equation 1a, MAP is also closely associated with SBP. Although there is a theory that the area under the curve of the systolic wave of pulse contour can be correlated with stroke volume and impedance coefficient (Saugel et al., 2021), there is still no direct equation to reflect the relationship between SBP and CO. Some studies have illustrated that SBP may be closer to the condition of tissue perfusion and patients’ outcomes. A study on 41 Spanish emergency departments among 10,979 patients suffering from acute heart failure demonstrated that 30-day mortality was negatively correlated with initial SBP. After adjustment for the risk factors, the prognostic impacts of hypo-perfusion on 30-day mortality varied across SBP categories (Rossello et al., 2021). Another clinical trial also indicated that SBP may be more related to patients’ tissue perfusion and outcomes perioperatively (Futier et al., 2017). Since the above paragraph has discussed that MAP may be not correlated well with CO and perfusion, is SBP more superior to MAP to reflect tissue perfusion and blood flow? Considering the influencing factors of SBP, SV, and aorta compliance, we apply the following function to express the influencing factors of SBP (Figure 1A):


(1c) SBP=(SV•AR)

AR refers to aorta resistance.

AR is determined by diastolic blood pressure and aortic wall compliance. On the condition that there is no established equation, we use the function (f) to express the uncertain relation among them mathematically, which can be expressed as:


(2a) AR=f⁢(DBP,Caorta).

AR is positively correlated with DBP, which is determined by stroke volume, heart rate, and peripheral vascular resistance. As a result,


(2b) $$\text{DBP}=f^{\prime }\,\left(\mathrm{SV},\mathrm{HR},\mathrm{SVR}\right)$$

In Function 2b, f′ stands for a function mathematically and represents the uncertain relation among DBP and SV, HR, and SVR. DBP is positively correlated with SV.

In equation 1c, we can substitute AR to function 2a:


(2c) SBP=SV•f⁢(DBP,Caorta)

Then, the composition of function 2b and 2c means that the domain of function 2b is in the domain of function 2c:


(2d, Figure 1B) $$\text{SBP}=\mathrm{SV}•f\,\left[f^{\prime }\,\left(\mathrm{SV},\mathrm{HR},\mathrm{SVR}\right),\mathrm{Caorta}\right]$$

Now we validate the relationship between MAP and SV, SBP, and SV in a mathematical way:

When HR and SVR are constants, according to Equation 1b, MAP is affected by SV once. On the other hand, from Function 2d, SBP should be estimated with a much higher contribution of SV, which is SV multiplied by SV. Therefore, any situation that causes the decrease of SV will cause the decrease of SBP, which is much more significant than MAP.

Increased HR can be compensated partly by decreased DBP, so what if SV decreases as HR increases at the same time? It can be further explained by the analysis of arterial pressure waveform: SBP can also more significantly reflect the changes of SV than MAP.

We assumed a clinical scenario: The initial MAP was 80 mmHg, SV was 70 ml, and HR was 70 bpm. MAP = DBP + 1/3 × (SBP −DBP), and DBP = 2/3 SBP.

SBP was assumed to be 103 mmHg and DBP 68 mmHg temporarily. When SV decreased from 70 to 35 ml, and HR increased from 70 to 140 bpm, MAP remained unchanged.

After HR increased, the role of SBP in MAP gradient increased and the role of DBP decreased. MAP would be closer to the value of SBP. Therefore, SBP decreased and was less than 103 mmHg in the presence of unchanged MAP. Heart rate increased, and cardiac output remained unchanged. Diastolic duration and blood flow decreased and DBP increased more than 68 mmHg (Figure 1C). In many cases, HR cannot be fully compensated and SVR also increased. Based on the principle that compensation cannot completely exceed the basic blood pressure, MAP will decrease but SBP will decrease more significantly (Figure 1D). So, we can conclude that SBP can sensitively reflect the change of SV and MAP can sensitively reflect the change of CO (SV multiplied by HR).

## The Decrease of SV Reflects the Early Hemodynamic Changes. Especially When CO Remains Unchanged, SBP Can Reflect Early Hemodynamic Changes

The common hemodynamic impairments in the perioperative period are usually caused by hypovolemic, distributive, cardiogenic, and obstructive factors (Vincent and De Backer, 2013; Noel-Morgan and Muir, 2018). During the perioperative period, hypovolemia after hemorrhage is the most common hemodynamic change. Due to the decreased blood volume and insufficient cardiac filling after hemorrhage, SV will decrease (Gruartmoner et al., 2015; Boissier et al., 2020; Shaylor et al., 2020). The subsequent body maintains blood pressure by adjusting sympathetic tension, increased cardiac contractility, and increased HR to compensate CO. However, the compensation could hardly cover the preliminary pathophysiological changes. The initial decrease of SV is also reported in other patients with obstructive and cariogenic hypotension (Hurewitz et al., 1986; Kearns and Walley, 2018; Hori et al., 2020). According to Figure 1, the decrease of SV is sensitively reflected in the change of SBP. Even if the increase of HR and SVR can leave CO unchanged, it will also lead to the decrease of SBP.

## Pulsatile Perfusion Is Beneficial to Organ Perfusion. When MAP Remains the Same, Lower SBP May Be Not Beneficial to Organ Perfusion

**FIGURE 2:**
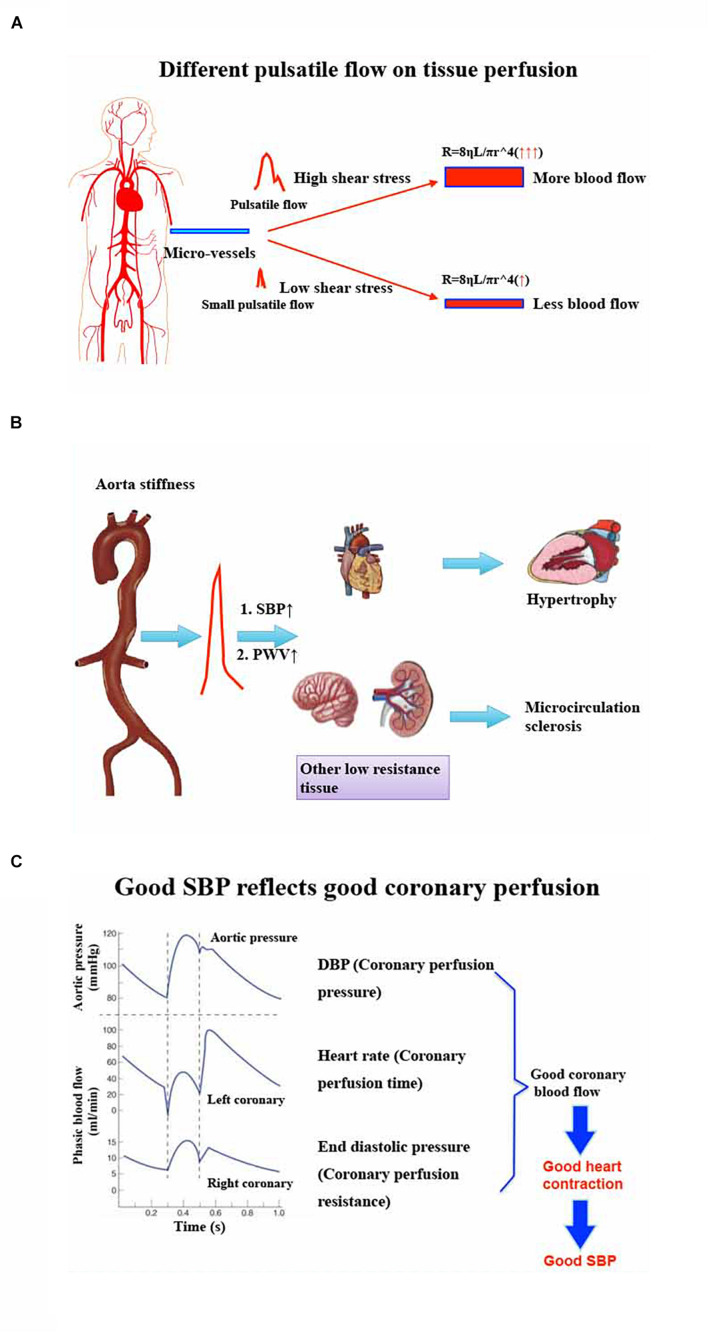
**(A)** Different pulsatile flow on tissue perfusion. Significant pulsatile blood flow will produce more shear stress to dilate micro-vessels. According to Poiseuille’s Law: R (Resistance) = 8ηL/πR∧^4^, these dilated micro-vessels will have more blood flow. However, small pulsatile blood flow will be difficult to dilate micro-vessels and, as a result, these vessels will have less blood perfusion. **(B)** Effect of aorta stiffness on target organs’ injury and hemodynamic management policy perioperatively. Aortic stiffness will produce higher SBP and higher PWV, which can cause myocardial hypertrophy and other low resistance organs’ (brain, kidney, etc.) micro-circulation sclerosis. So, the heart may need higher DBP and other organs might need higher MAP or SBP to perfuse them perioperatively. **(C)** Good SBP reflects good coronary perfusion. The typical theory informs us that the coronary perfusion depends on DBP because the ventricular wall tension, especially the left ventricular wall tension, is lower during the diastolic phase. However, good DBP does not represent good coronary perfusion because heart rate and ventricular end diastolic pressure will influence coronary perfusion by interfering with diastolic duration and coronary microcirculation resistance. Good SBP may reflect good heart contraction, which is regarded as the result of enough coronary blood flow. SBP, systolic blood pressure. DBP, diastolic blood pressure. PWV, pulse wave velocity. MAP, mean artery pressure.

According to the formula of Poiseuille’s Law, R (Resistance) = 8ηL/πR∧^4^, vessels resistance is closely related to viscosity, vessel diameter, and vessel length (Pfitzner, 1976). No matter whether SBP or SV stay the same or not, as long as MAP and CO are the same, organ blood flow will be negatively correlated with vascular resistance (Holmes et al., 2020). During cardiopulmonary bypass, pulsatile blood flow could provide more blood flow in the brain and other organs compared with non-pulsatile blood flow (Murkin and Farrar, 1989; Serraino et al., 2012; Milano et al., 2015). It might be that more pulsatility will produce larger shear force to dilate small arteries and result in more blood flow (Sicsic et al., 1998; Nakano et al., 2000; Koning et al., 2012). When MAP is the same, lower SBP means lower pulsatility. Meanwhile, lower pulsatility may produce smaller shear force and yield lower organ blood flow. In the elderly with high SBP due to arteriosclerosis, the compliance of microcirculation dilation becomes poor because of vascular remodeling. These patients often need higher MAP to obtain satisfactory blood flow, or higher shear force (higher SBP) to keep small vessels open perioperatively (Figure 2A).

## Patients With High Systolic Blood Pressure Before Operation Need Higher Perfusion Pressure During Perioperative Period. A Significant Decrease of Systolic Blood Pressure Should Be Avoided

In high SBP patients, the increase of blood pressure pulsation and pulse wave velocity (PWV) can result in arteriosclerosis, remodeling, and expansibility, especially for the heart, brain, kidney, and other organs with low vascular resistance (Ben-Shlomo et al., 2014; Chirinos et al., 2019). Although higher SBP before operation can promote microcirculation sclerosis, any factor that causes the decrease of SBP during the perioperative period may cause organ blood supply insufficiency. In terms of this kind of patient, perioperative low SBP should be avoided. In recent years, it has been reported that organ function can be better maintained by maintaining high SBP that is assumed to be closer to the basic SBP (Futier et al., 2017), which can be explained further by Figure 2B.

## DBP Represents Coronary Artery Perfusion Pressure Rather Than Coronary Artery Blood Flow. Perfusion Pressure Is Not Equal to Blood Flow. Good Systolic Blood Pressure May Represent Good Coronary Blood Flow Better Than DBP or MAP

MAP is related to the blood supply of heart, brain, and kidney, especially for the brain (Meng et al., 2015). However, for the heart, the coronary perfusion is more dependent on DBP. In addition, the increase of HR and left ventricular wall tension may affect the coronary blood flow in spite of good DBP (Goodwill et al., 2017). Therefore, the decrease of SV accompanied by the increase of HR, or the use of drugs to increase SVR, seems to be able to maintain MAP and DBP well, while the coronary blood flow may be significantly reduced. Reduced coronary blood flow may influence myocardial contractility and yield low SBP, especially in the early period, which is more obvious for elderly coronary artery disease patients. There will be a vicious circle of low blood pressure and coronary blood flow. Therefore, good DBP may provide good coronary artery perfusion pressure but does not represent good coronary blood flow. SBP is more correlated with good systolic function and could reflect good coronary blood flow when compared with DBP or MAP. Although there seems no direct evidence to compare SBP and DBP on coronary blood flow, a relatively normal SBP may provide both good coronary blood flow and other organs’ perfusion. Furthermore, avoiding low SBP is also a direct way to prevent myocardial injury during perioperative time (Abbott et al., 2018). If we pay more attention to SBP perioperatively and can more positively deal with abnormal low SBP even if MAP is not changed, we may improve the hemodynamics in the early period and prevent some hemodynamic or cardiovascular vicious circle (Figure 2C).

## Blood Pressure and Tissue Perfusion in Atrial Fibrillation and Other Arterial Wall Stiffness Patients

Due to the lack of effective atrial systole, for atrial fibrillation patients, stroke volume may be reduced (Kotecha and Piccini, 2015; Kallistratos et al., 2018). Also, stroke volume or cardiac output may be not significantly reduced because of preserved ejection fraction or increased heart rate which can partly compensate for inadequate ventricular filling (Lewis et al., 1988; Sartipy et al., 2017). As a result, there is a possibility that neither stroke volume nor cardiac output will decrease remarkably. However, for rapid atrial fibrillation, the above hemodynamic compensation effects cannot overcome inadequate ventricular filling (Clemo et al., 1998). Therefore, significant hypotension or hypo-perfusion may happen. In circumstances of other irregular rhythms, SBP may vary frequently and cardiac output may be more stable to evaluate cardiac output and tissue perfusion. However, the mathematic hemodynamic model may be more complicated and need to be explored further.

Arterial wall stiffness may be significant with aging or other diseases, including diabetics and morbid obesity (Mikael et al., 2017; Durham et al., 2018). It needs higher blood pressure to perfuse the tissues in the presence of prior arterial wall stiffness. The ideal beneficial therapy is to reduce arterial wall stiffness. Maintaining a higher blood pressure or higher SBP is a temporary alternative method to maintain tissue perfusion, especially during perioperative time. What’s more, compared with MAP, SBP can provide pulsatile flow with more shear stress to dilate the stiff arterial wall, even if prolonged higher blood pressure may deteriorate arterial wall stiffness (Li et al., 2003; Mitchell, 2014; Morales-Acuna et al., 2019). According to the function 2d: SBP = SVf [f’ (SV, HR, SVR), Caorta] and function 1b: MAP = SVHRSVR, arterial wall stiffness could increase the value of both SVR and Caorta. SBP will be influenced doubly by stroke volume while MAP will be influenced only singly by stroke volume. We can easily conclude that, when stroke volume changes, the change of SBP is more significant than MAP. Meanwhile, maintaining a relatively normal SBP may promote anesthesiologists to correct early hemodynamic changes during surgery and can maintain enough tissue perfusion.

In conclusion, MAP can sensitively reflect cardiac output change and SBP can sensitively reflect stroke volume change as well. Moreover, SBP can reflect the early hemodynamic changes, organs’ perfusion, and heart systolic function. Compared with MAP, perioperative monitoring of SBP and timely detection and treatment of abnormal SBP are very important for the early detection of hemodynamic abnormalities (Table 1).

**TABLE 1:** Differences of MAP and SBP in reflecting perfusion and hemodynamics.

	MAP	SBP
Reflect early hemodynamic changes	No	Strong
Reflect cardiac output	Linear correlation when SVR fixed	Correlation (not linear)
Reflect stroke volume	Poor	Strong
Reflect organs perfusion	Moderate	Strong
Reflect heart systolic function	Poor	Strong

## Declarations

The writing and submission of this report were approved by all the authors who agreed to submit this manuscript for possible publication.

## Author Contributions

JS and JY wrote the manuscript. BL finalized and revised the manuscript. All authors contributed to the article and approved the submitted version.

## Conflict of Interest

The authors declare that the research was conducted in the absence of any commercial or financial relationships that could be construed as a potential conflict of interest.

## Publisher’s Note

All claims expressed in this article are solely those of the authors and do not necessarily represent those of their affiliated organizations, or those of the publisher, the editors and the reviewers. Any product that may be evaluated in this article, or claim that may be made by its manufacturer, is not guaranteed or endorsed by the publisher.
